# Highly sensitive chemiluminescent aptasensor for detecting HBV infection based on rapid magnetic separation and double-functionalized gold nanoparticles

**DOI:** 10.1038/s41598-018-27792-5

**Published:** 2018-06-21

**Authors:** Zhijiang Xi, Quan Gong, Chao Wang, Bing Zheng

**Affiliations:** 1 Department of Clinical Laboratory, The Second Clinical Medical College, Yangtze University, Jingzhou, 434020 China; 2 School of Medicine, Yangtze University, Jingzhou, 434023 China

## Abstract

Hepatitis B virus (HBV) infection is a major global public health problem and one of the leading causes of chronic liver disease. HBsAg is the first serological marker to appear in the blood and is the most important marker of HBV infection. Detection of HBsAg in serum samples is commonly carried out using an immunoassay such as an enzyme-linked immunosorbent assay (ELISA), which is complex to perform, time-consuming, and unsatisfactory for testing sensitivity. Therefore, new methods for highly sensitive detection of HBV infection are urgently needed. Aptamers are specific recognition molecules with high affinity and specificity toward their targets. Biosensors that employ aptamers as biorecognition elements are known as aptasensors. In this study, we select an HBsAg-specific aptamer and use it to develop a new chemiluminescent aptasensor based on rapid magnetic separation and double-functionalized gold nanoparticles. This sensor enables rapid magnetic separation and highly sensitive detection of HBsAg in HBV-positive serum. The detection limit of this HBsAg-detecting chemiluminescent aptasensor is as low as 0.05 ng/mL, which is much lower than the 0.5 ng/mL limit of a typical ELISA used in hospitals. Furthermore, this aptasensor works well and is highly specific to HBV infection.

## Introduction

Hepatitis B virus (HBV) is a partially double-stranded DNA virus that is highly infectious and prevalent among all world populations. HBV infection, a major global public health problem and one of the leading causes of liver disease, is the most common chronic viral infection^1–3^. An estimated 2 billion people worldwide have been infected with HBV, 240 million of whom are chronically infected, with a 5–20% risk of developing chronic liver diseases such as chronic hepatitis, cirrhosis, hepatic decompensation, and hepatocellular carcinoma, especially in developing countries^4–7^. Furthermore, approximately 780,000 patients die from advanced liver diseases caused by chronic HBV infection each year^8^.

There are several serological markers for HBV infection: hepatitis B surface antigen (HBsAg), hepatitis B surface antibody (HBsAb), hepatitis B e antigen (HBeAg), hepatitis B e antibody (HBeAb), and hepatitis B core antibody (HBcAb). Among these markers, HBsAg is the first to appear in the blood and is the most important marker of HBV infection. Therefore, the detection of HBsAg is a significant tool for diagnosing an HBV infection^9–11^.

HBsAg can be detected with available assay technology before clinical hepatitis develops—typically as early as 6–8 weeks after infection. There is a continuing need to develop more sensitive assays, to shorten the period between infection and the detection of infection markers and produce easy-to-perform assays for daily screening. Detection of HBsAg in serum samples is commonly carried out using an immunoassay such as an enzyme-linked immunosorbent assay (ELISA), which is currently the most appropriate approach to clinical biomarker detection; it is specific, inexpensive, and has a straightforward readout^12^. However, ELISA is complex to perform, time-consuming, and unsatisfactory for testing sensitivity, which severely hampers its wider application in clinical detection^13^. Therefore, it is important to seek highly sensitive new methods for detecting HBV infection.

**Figure 1:**
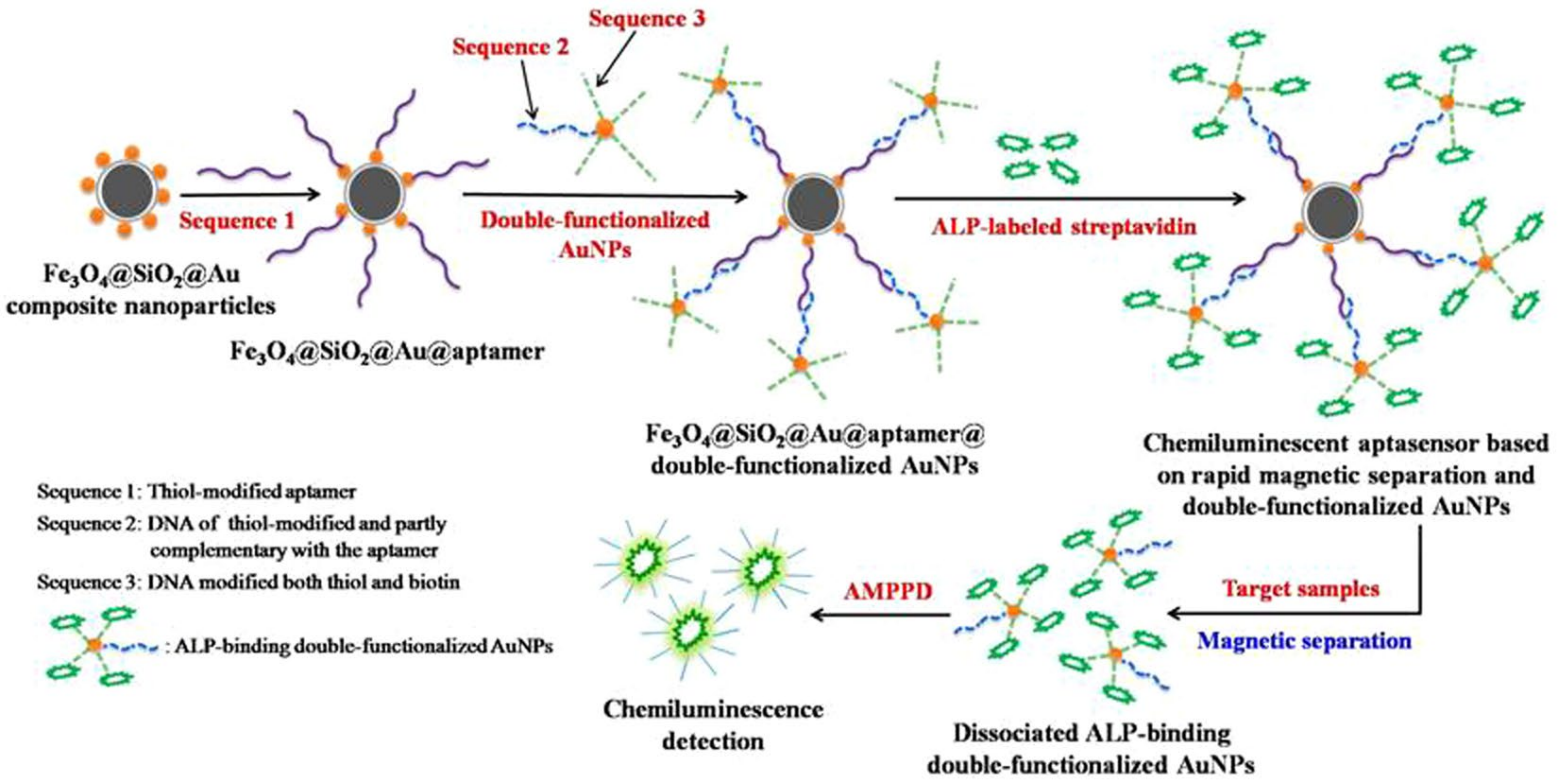
Schematic illustration of the chemiluminescent aptasensor based on rapid magnetic separation and double-functionalized gold nanoparticles.

Aptamers are short single-stranded oligonucleotides selected by an *in vitro* technology known as the systematic evolution of ligands by exponential enrichment (SELEX). Aptamers are a new type of specific recognition molecules and are viewed as artificial antibodies, but they possess several advantages when compared with antibodies: they are very stable, non-immunogenic and non-toxic, simple to chemically modify, and high binding affinity and specificity toward their targets. Biosensors that utilize aptamers as biorecognition elements are known as aptasensors, and have been broadly applied to the detection of targets including proteins, microorganisms, viruses, and cancer cells^14–16^. We have reported the successful selection of aptamers against HBsAg based on magnetic nanoparticles (MNPs), which were used as separated medium^17^. The aim of this study is to improve the method’s sensitivity and simplify its operation for detecting HBV infection. We select an HBsAg-specific aptamer and use it to develop a new chemiluminescent aptasensor based on rapid magnetic separation and double-functionalized gold nanoparticles (AuNPs), which allows for rapid magnetic separation and highly sensitive detection of HBsAg in HBV-positive serum. The new chemiluminescent aptasensor was developed as shown in Fig. 1. Here, the gold magnetic composite nanoparticles (Au@Fe_3_O_4_@SiO_2_ NPs) are used to immobilize an HBsAg-specific aptamer for rapid magnetic separation; the detection signal can then be amplified by double-functionalized gold nanoparticles.

## Results

### Optimization of detection conditions

**Figure 2:**
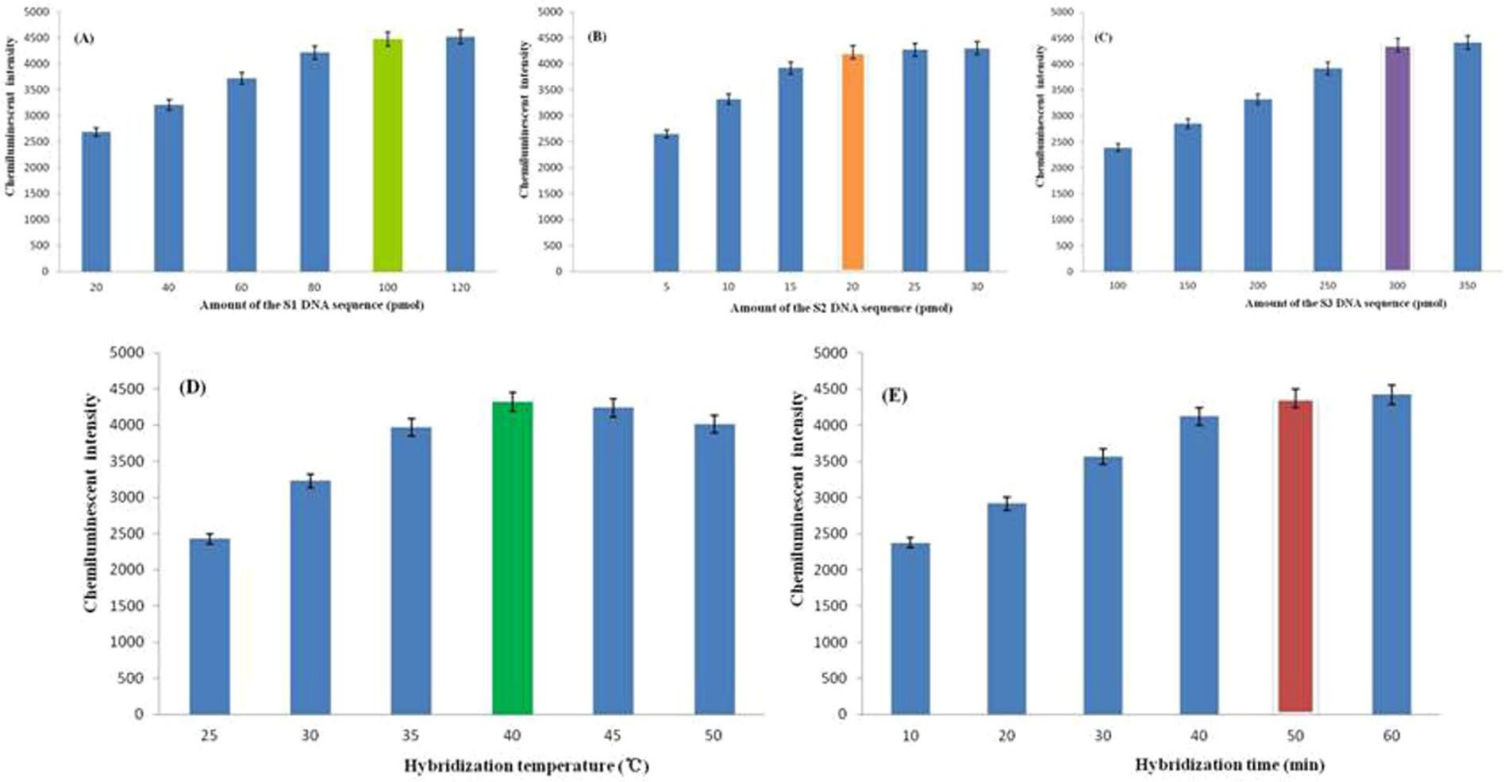
Optimization of the conditions for detecting HBsAg, including amount of the S1 DNA sequence, amount of the S2 DNA sequence, amount of the S3 DNA sequence, hybridization temperature between S1 and S2, and hybridization time between S1 and S2.

A thiol-modified aptamer against HBsAg (referred to as Sequence 1, abbreviated as S1), a sequence modified with thiol and partly complementary with the aptamer against HBsAg (referred to as Sequence 2, abbreviated as S2), and a sequence modified with both thiol and biotin (referred to as Sequence 3, abbreviated as S3) were used in this study. Conditions for HBsAg detection were optimized (Fig. 2), including amount of the S1, S2, and S3 DNA sequence, respectively; and hybridization temperature and hybridization time between S1 and S2. Figure 2A shows that chemiluminescent intensity gradually increased as the amount of S1 increased, but this increase slowed substantially for amounts of 100 pmol or greater. Therefore, considering the cost of aptamer synthesis, we determined 100 pmol to be the optimal amount of S1. As shown in Fig. 2B,C, chemiluminescent intensity first increased and then decreased with increasing amounts of S2 and S3. Based on these results, the optimal amounts of S2 and S3 selected were 20 pmol and 300 pmol, respectively, considering synthesis costs.

Chemiluminescent intensity increased and then decreased as the hybridization temperature between S1 and S2 increased; the optimum temperature was 40 °C (Fig. 2D). As shown in Fig. 2E, chemiluminescent intensity gradually increased as hybridization time increased; the increase slowed substantially at 50 min. Based on this finding, the optimal hybridization time between S1 and S2 was set to 50 min.

### Detection of pure HBsAg protein

**Figure 3:**
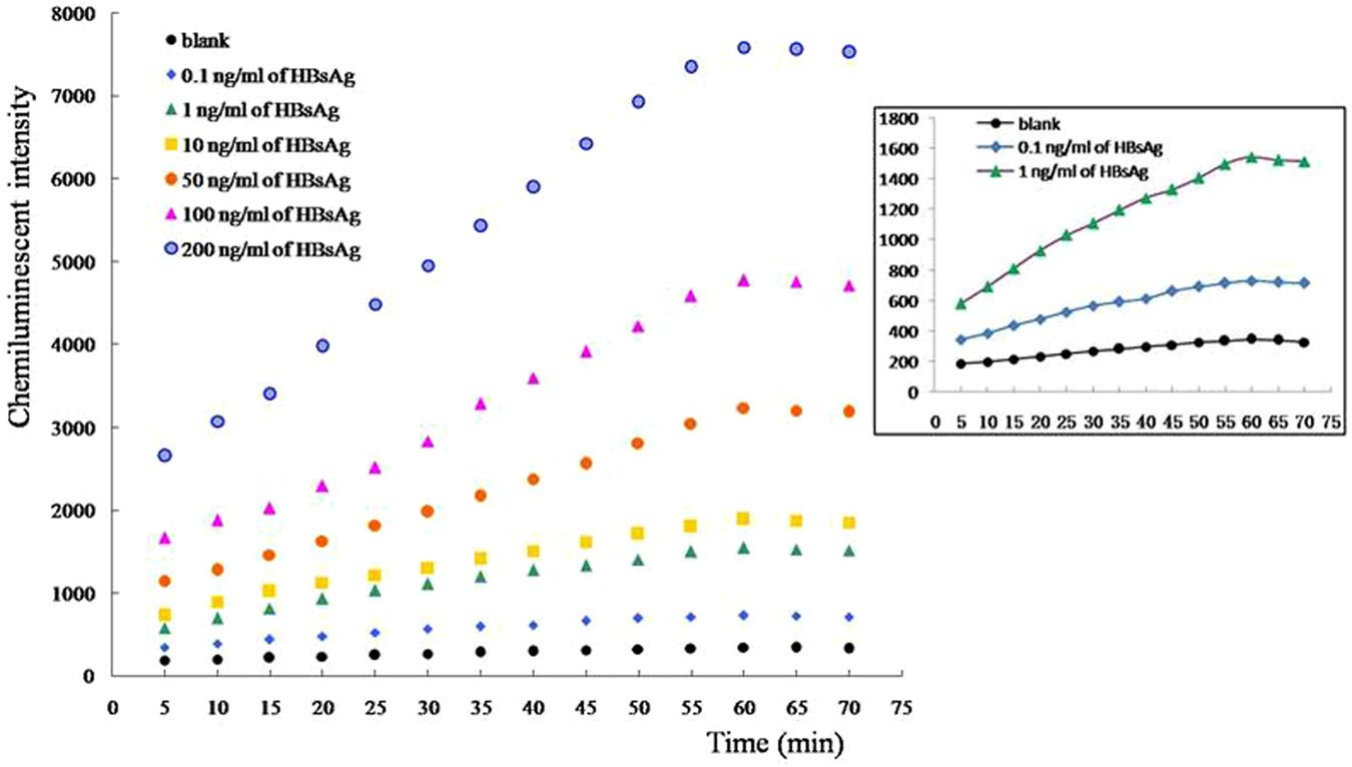
The chemiluminescent intensity for different concentrations of pure HBsAg protein using our chemiluminescent aptasensor.

**Figure 4:**
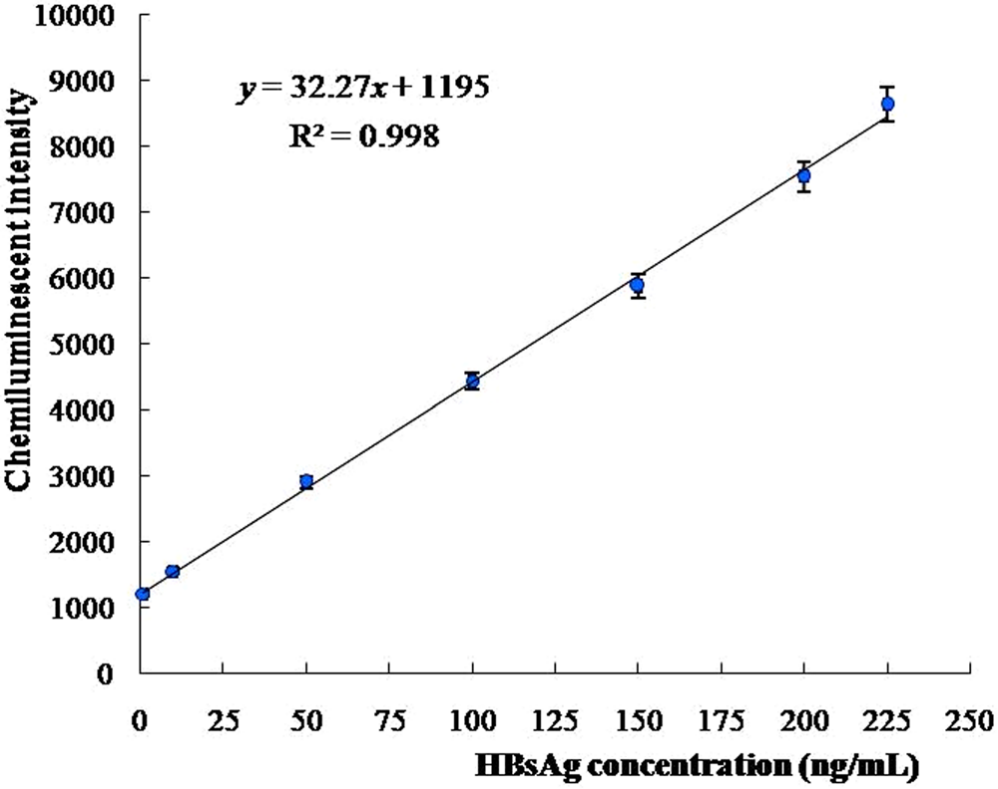
The linear relationship between chemiluminescent intensity and HBsAg concentration in the range of 1–225 ng/mL.

Various concentrations of pure HBsAg protein were prepared in several dilutions, and were detected using our newly developed chemiluminescent aptasensor. As shown in Fig. 3, chemiluminescent intensity gradually increased with HBsAg concentration. Furthermore, there was a linear relationship between chemiluminescent intensity and HBsAg concentration in the range of 1–225 ng/mL (Fig. 4). The regression equation for this relationship was *y* = 32.27*x* + 1195, with a regression coefficient of 0.998.

### Detection of HBsAg in actual serum samples

**Figure 5:**
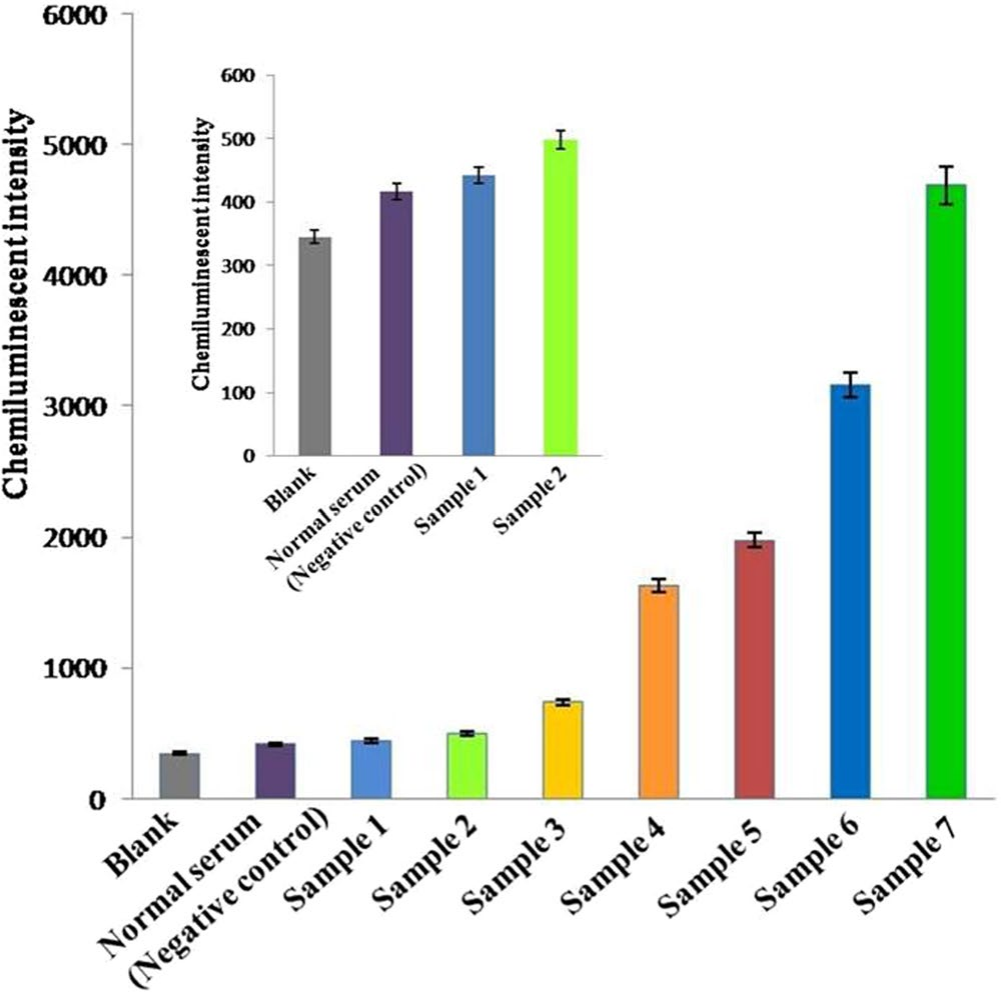
Detection results for seven HBV-positive serums; the aptasensor’s limit for detecting HBsAg is as low as 0.05 ng/mL.

Thirty serum samples were tested with our newly developed chemiluminescent aptasensor. The results were roughly in line with ELISA results; both deviations were less than 5%—ranging from 0.51% to 4.29%—indicating the feasibility of this detection system for actual serum samples. Figure 5 shows detection results for seven HBV-positive serums. The HBsAg concentrations were 0.01, 0.05, 0.1, 1, 10, 50, and 100 ng/mL. We estimated the detection limit of HBsAg to be 0.05 ng/mL (*Q* = 2.15 > 2.1), which is much lower than the 0.5 ng/mL limit of the ELISA currently in clinical use.

### Specificity of the chemiluminescent aptasensor toward HBV-positive serum

**Figure 6:**
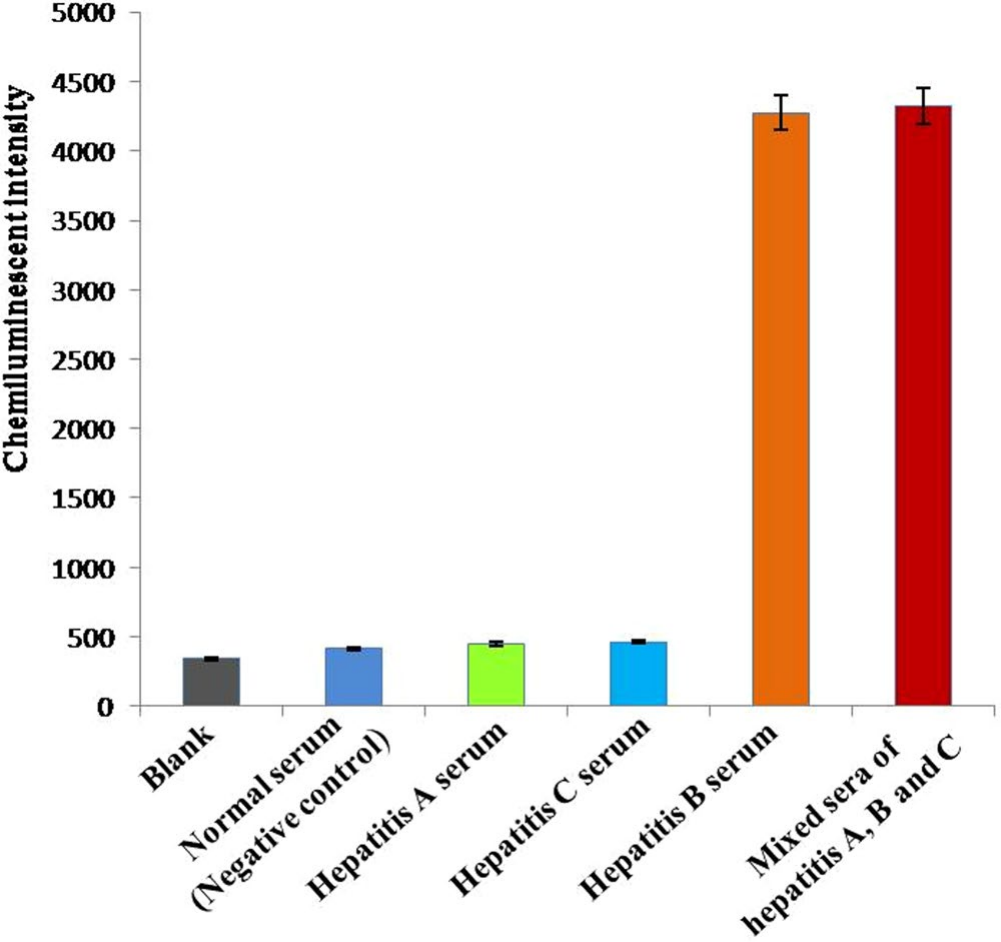
The specificity of the chemiluminescent aptasensor toward HBV-positive serum (hepatitis **B** serum) and other control serum samples including normal serum, hepatitis (**A**) serum, hepatitis (**C**) serum, and mixed sera of hepatitis (**A**–**C**).

To evaluate the specificity of the chemiluminescent aptasensor toward HBV-positive serum containing HBsAg, we conducted experiments on the following controls: normal serum, hepatitis A serum, hepatitis C serum, and a mixed sera of hepatitis A, B and C. The result shown in Fig. 6 demonstrated that the chemiluminescent signals of normal serum, hepatitis A serum, and hepatitis C serum were very low, close to that of a blank control. However, a significant increase in chemiluminescent intensity was observed on exposure to HBV-positive serum, indicating that our chemiluminescent aptasensor was highly specific to the target serum. The cross-sensitivity of this aptasensor was also examined for a mixture of the 3 different serums. Compared with the results of hepatitis B serum, no significant change was observed in the mixed sera of hepatitis A, B and C, which indicated that hepatitis A serum and hepatitis C serum had a negligible influence on the detection of hepatitis B serum. Therefore, even in the presence of interfering substances such as co-infections, this chemiluminescent aptasensor worked well and had high specificity toward HBV infection.

## Discussion

Immunoassays such as ELISA are very reliable and are widely used for detecting disease biomarkers via antibody–antigen interactions. However, ELISA has the disadvantage of insufficient sensitivity. Therefore, new methods for highly sensitive detection of disease biomarker, including for all kinds of HBV-positive serum markers, are urgent. Zhang *et al*. developed simple, sensitive fluorescent detection of HBsAg using a dot-blot immunoassay with quantum-dot nanobeads (QDNBs) for signal amplification^12^; the detection limit of the immunoassay was greatly improved by QDNBs, and could detect as little as 78 ng/mL of HBsAg protein immobilized on the hydrophobic polyvinylidenedifluoride membrane.

Aptamers have high binding affinity and specificity toward their recognized targets, and aptasensors are a new type of biosensors that use aptamers as biorecognition elements. Chemiluminescence methods have various advantages, such as a wide linear dynamic range, simplified operation, easy automation, and high sensitivity. Therefore, chemiluminescent aptasensor have attracted increasing attention. We have reported a chemiluminescent aptasensor based on Fe_3_O_4_ MNPs and an immunoassay for rapid detection of HBsAg with a detection limit 0.1 ng/mL^17^. However, Fe_3_O_4_ MNPs have a masking effect on chemiluminescence, which reduces detection sensitivity. To solve this issue, we developed another chemiluminescent aptasensor based on rapid magnetic separation and double-functionalized gold nanoparticles, in which ALP-labeled streptavidin was immobilized on the AuNPs not on Fe_3_O_4_ nanoparticles to catalyze the chemiluminescence. The Fe_3_O_4_@SiO_2_@Au composite nanoparticles were used to immobilize HBsAg-specific aptamer for rapid magnetic separation; this detection signal can then be amplified by AuNPs, which can bind with ALP-labeled streptavidin to catalyze the chemiluminescence and mitigate the masking effect of Fe_3_O_4_ nanoparticles on chemiluminescence.

In this study, an HBsAg-specific aptamer was used to develop a chemiluminescent aptasensor based on rapid magnetic separation and double-functionalized gold nanoparticles, which allowed rapid magnetic separation and highly sensitive detection of HBsAg in HBV-positive serum. The detection limit of this new chemiluminescent aptasensor for the detection of HBsAg was as low as 0.05 ng/mL, which is much lower than the 0.5 ng/mL limit of an ELISA. Furthermore, this chemiluminescent aptasensor worked well and had high specificity toward HBV infection, even in the presence of interfering substances such as co-infections.

## Methods

### Ethics statement

All experiments were carried out in accordance with relevant guidelines and regulations. The study was approved by the Ethics Committees of School of Medicine, Yangtze University. Informed consent of the study subjects was not required due to the nature of the samples.

### Reagents and materials

Chloroauric acid (HAuCl_4_), alkaline phosphatase (ALP)-labeled streptavidin, and 3-(2′-spiroadamantane)-4-methoxy-4-(3′′-phosphoryloxy)-phenyl-1,2-dioxetane (AMPPD) were all purchased from Shanghai Sangon Biotech Co. Ltd. (Shanghai, China). 3-mercaptopropyl triethoxysilane (MPTES) was purchased from Sigma–Aldrich, Inc. (USA). HBsAg was purchased from Shanghai Chao Yan Bio-technology Ltd. (Shanghai, China). Ethylene glycol, iron chloride hexahydrate (FeCl_3_·6H_2_O), tetraethyl orthosilicate (TEOS), and citrate sodium were all purchased from Nanjing Chemical Reagent Ltd. (Nanjing, China). The other chemical reagents were all analytical grade from Nanjing Ronghua Reagent Ltd. (Nanjing, China). Water used in the experiments was deionized prior to use. All serum samples were kindly provided by the Second Hospital of Nanjing, China.

Sequence 1 containing a 40-base central random sequence flanked by primer sites on either side, Sequence 2, and Sequence 3 were all synthesized by Shanghai Sangon Bioltech Co. Ltd. The above sequences are as follows:

Central 40 bases of Sequence 1: 5′-CACAGCGAACAGCGGCGGACATAATAGTGCTTACTACGAC-3′

Sequence 2: 5′- CGAGCTCGAATTCCCGATCTCTAG-SH-3′

Sequence 3: 5′- Biotin-TCGCAGTGT-SH-3′.

### Preparation of AuNPs and Au@Fe_3_O_4_@SiO_2_ NPs

We prepared all nanoparticles in this study according to our previously published articles. The Fe_3_O_4_ MNPs were synthesized via a modified solvothermal method^18–20^. Then, the prepared Fe_3_O_4_ MNPs were coated with silica using a modified Stöber method to improve the dispersity of the Fe_3_O_4_@SiO_2_ nanoparticles. The AuNPs were prepared by the citrate sodium reduction method^19^. The thiol-modified Fe_3_O_4_@SiO_2_ nanoparticles were combined with AuNPs via self-assembly to form Au@Fe_3_O_4_@SiO_2_ NPs.

### Construction of a new chemiluminescent aptasensor

We selected aptamers against HBsAg successfully using SELEX technology *in vitro* based on carboxylated MNPs. The new chemiluminescent aptasensor based on rapid magnetic separation and double-functionalized gold nanoparticles was developed as follows. First, the thiol-modified aptamer against HBsAg (Sequence 1, S1) was incubated with the Fe_3_O_4_@SiO_2_@Au NPs to form an Fe_3_O_4_@SiO_2_@Au@aptamer composite. Second, double-functionalized gold nanoparticles modified with two kinds of DNA sequences were prepared. Sequence 2 (S2) was modified with thiol and was partly complementary with the aptamer against HBsAg; Sequence 3 (S3) was modified with both thiol and biotin. Third, the composite Fe_3_O_4_@SiO_2_@Au@aptamer was hybridized with double-functionalized gold nanoparticles (S2-AuNPs-S3) to form an Fe_3_O_4_@SiO_2_@Au@aptamer@S2-AuNPs-S3 composite. Finally, ALP-labeled streptavidin was added to incubate, thus producing a new chemiluminescent aptasensor based on rapid magnetic separation and double-functionalized gold nanoparticles. When the target HBsAg was added to the chemiluminescent aptasensor, the ALP-binding double-functionalized AuNPs (S2-AuNPs-S3-ALP) dissociated from the aptamer due to competitive specific binding of the target to the aptamer, which was used subsequently to catalyze the chemiluminescence of the AMPPD substrate. Chemiluminescence was detected with an Enspire 2300 multilabel reader (PerkinElmer, USA).

### Optimization of detection conditions

We optimized the conditions used for detecting HBsAg, including amount of the S1 DNA sequence, amount of the S2 DNA sequence, amount of the S3 DNA sequence, hybridization temperature between S1 and S2, and hybridization time between S1 and S2. The amounts of S1 tested were 20, 40, 60, 80, 100, and 120 pmol, and the amounts of S2 investigated were 5, 10, 15, 20, 25, and 30 pmol. The double-functionalized AuNPs were prepared such that both thiol-modified S2 and S3 were combined with AuNPs via the covalent binding of Au-S bonds. The ALP-labeled streptavidin catalyst was combined with S3-labeled biotin to form the composite catalyst S2-AuNPs-S3-ALP, which was used to catalyze the chemiluminescence of AMPPD during detection. To amplify the detection signal of the composite catalyst S2-AuNPs-S3-ALP for the chemiluminescence of AMPPD, the amount of S3 was increased several times proportionally than that of S2, to 100, 150, 200, 250, 300, and 350 pmol. We tested S1-S2 hybridization temperatures of 25, 30, 35, 40, 45, and 50 °C and hybridization times of 10, 20, 30, 40, 50, and 60 min.

### Testing sensitivity and specificity

The sensitivity and specificity of a biosensor are very significant, and determine whether the biosensor can be used in practical applications. The sensitivity of our newly developed chemiluminescent aptasensor was tested on pure HBsAg protein and HBV-positive serum containing HBsAg. Furthermore, the sensor’s specificity for HBV-positive serum was also assessed using normal serum, hepatitis A serum, hepatitis C serum, and a mixed sera of hepatitis A, B, and C as controls. Each test used 40 μL of each of the samples. Each experiment or treatment was repeated 3 times.

### Data availability

All data generated or analysed during this study are included in this published article.
